# Non-exercise based estimation of cardiorespiratory fitness is inversely associated with metabolic syndrome in a representative sample of Korean adults

**DOI:** 10.1186/s12877-020-01558-z

**Published:** 2020-04-19

**Authors:** Inhwan Lee, Shinuk Kim, Hyunsik Kang

**Affiliations:** 1 College of Sport Science, Sungkyunkwan University, Suwon, Republic of Korea; 2 College of Kyedang General Education, Sangmyung University, Cheonan, South Korea; 3 Laboratory of Exercise Physiology and Biochemistry, College of Sport Science, Sungkyunkwan University, 2066 Seobu-Ro, Jangan-Gu, Suwon, 16419 Republic of Korea

**Keywords:** Physical fitness, Lifestyle, Gender, Metabolic complications, Korean adults

## Abstract

**Background:**

This study investigated the association between non-exercise based estimation of cardiorespiratory fitness (eCRF) and metabolic syndrome (Mets) in Korean adults aged 18 years and older (13,400 women and 9885 men).

**Methods:**

Data from the 2008 and 2011 Korea National Health and Nutrition Examination Surveys IV and V in South Korea were analyzed. eCRF was assessed with a previously validated procedure. Participants were classified into 5 categories from the lowest quantile to the highest quantile based on individual eCRF distributions.

**Results:**

The findings showed an independent and inverse association between eCRF and Mets in women and men separately. Individuals in the highest eCRF category (quantile 5) had a significantly lower prevalence of Mets (14.5 and 14.8% for women and men, respectively) compared with their counterparts (40.4 and 46.4% for women and men, respectively) in the lowest eCRF category (quantile 1), and the association showed a graded response, with the quantiles 2, 3, and 4 also significantly associated with a lower prevalence of Mets compared with the quantile 1. Furthermore, the prevalence of Mets in the highest quantile compared with the lowest quantile remained statistically significant in both men (*p* < 0.05) and women (*p* < 0.05) even after adjustments for age, body mass index, skeletal muscle index, smoking, heavy drinking, vitamin D, caloric intake, and dietary intakes of carbohydrates, fats, and proteins.

**Conclusion:**

The findings support a preventive role for eCRF against Mets in Korean adults.

## Background

Metabolic syndrome (Mets) represents a clustering of risk factors such as central obesity, hyperglycemia, hypertension, hypertriglyceridemia, and decreased high-density lipoprotein cholesterol [1]. Left untreated, Mets leads to the development of non-communicable diseases, such as type 2 diabetes and cardiovascular diseases (CVDs) in residents of both developed and developing countries [2, 3]. Western lifestyles characterized by obesity and physical inactivity have been blamed for the etiology of the global epidemic [1]. Along with unhealthy lifestyles, low level of cardiorespiratory fitness (CRF), which reflects the maximal capacity of the respiratory and cardiovascular systems to supply oxygen to working skeletal muscles during exercise, is another well-established risk factor of Mets [4, 5]. Low CRF is also associated with an increased risk of all- and specific-cause mortality [6–8].

In Korea, a third of adults suffer from Mets. The prevalence of Mets is on the rise, particular in men, and in those of advancing age, with the greatest prevalence seen in elderly persons [9]. Considering continuing rise in prevalence and adverse consequences in conjunction with a rapidly aging society, Mets will continue to represent a key public health issue in Korea. Obesity and physical inactivity have been suggested as two of the primary lifestyle risk factors responsible for Mets in Korea [10]. However, less attention has been paid to investigating the impact of CRF for Mets in Korean populations. A literature review uncovered only 2 studies that examined physical fitness in relation to Mets in Korean adults. In a cross-sectional study involving 227 older adults aged 60 years and older, Hwang and Kim [11] examined the association between physical performances of a sit-up test and the Tecumseh step test as indices of muscular fitness and cardiopulmonary fitness, respectively, and they reported an inverse relationship between muscular and/or cardiopulmonary fitness and Mets. In another study involving 1007 Korean adults who underwent routine health checkups, Hong et al. [12] examined the association between a step test-based CRF and Mets, and they found that low CRF and obesity were significantly associated with an increased risk of Mets. Those previous studies recognized a prognostic role of CRF but failed to prove it in a representative sample of Korean adults. Consequently, the relationship of CRF with Mets in Korean populations remains to be confirmed.

Objectively measuring CRF is often limited by the need for specialized equipment, trained personnel, sufficient time, and other factors [8], especially in a population-based study involving a large sample size. To circumvent those limitations, Jurca et al. [13] showed that CRF can be estimated from routinely obtained health indicators with an acceptable accuracy. We previously showed that this non-exercise based estimation of CRF (eCRF) could be used an alternative tool to estimate the risk of morbidity and mortality from all and specific causes in Korean adults [14]. This study examined the association between eCRF and Mets in a representative sample of Korean adults aged 18 years and older.

## Methods

### Data source and study population

**Fig. 1:**
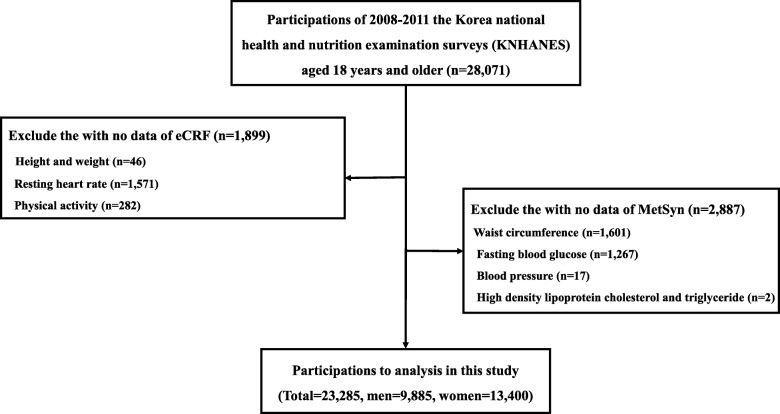
Flow of eligible participants in the study

The data used for this study were drawn from the Korea National Health and Nutrition Examination Surveys (KNHANES) IV and V, which were conducted nationwide in South Korea from 2008 until 2011. A detailed description of the KNHANES, including the sampling method, is available elsewhere [15, 16]. For the current study, we selected a total of 28,071 adults aged 18 years and older from those who participated in the 2008–2011 KNHANES IV and V. Of them, 1899 individuals were excluded because parameters (46 missing in height and weight, 1571 missing resting heart rate, and 282 missing in physical activity) used to estimate CRF were not available. An additional 2887 were excluded because the components of Mets (1601 missing in waist circumference, 1267 missing in fasting blood glucose, 17 missing in resting blood pressure, and 2 missing in blood lipids) were not available. Ultimately, 23,285 adults (13,400 women and 9885 men) were included in the final data analyses (Fig. 1). The Korean National Institute for Bioethics Policy reviewed and approved the study design (P01–201504–21-005) in accordance with the Declaration of Helsinki. Informed consent was obtained from all of the study. Written informed consent was obtained from all participants included in the study.

### Estimation of cardiorespiratory fitness

Non-exercise-based estimation of CRF (eCRF) was determined as peak volume of oxygen consumption (peak VO_2_) using a formula described by Jurca et al. [13]: eCRF (ml·kg^− 1^·min^− 1^) = 2.77 (men = 0 and women = 1) - 0.10 (age) - 0.17 (body mass index) - 0.03 (resting heart rate) + 1.00 (physical activity score) + 18.07.

Participants were then classified into five categories (from the lowest to the highest quantile) on the basis of age- and sex-specific quantiles of their estimated peak VO_2_ distributions.

### Clinical and laboratory measurements

Participants were required to complete self-administered questionnaires regarding smoking and alcohol habits, physical activity, and past medical history. Smoking status was classified as current smokers or non-smokers. Heavy drinking was defined as > 14 drinks/week for men and > 7 drinks/week for women on the basis of drinking frequency (days per week) and quantify (drinks per week) in the past year [17]. The Korean version of International Physical Activity Questionnaire (IPAQ) short form was used to assess frequency (times/week) and duration (minutes) of physical activity lasting for at least 10 min according to intensity (light, moderate, and vigorous, which were expressed in METs-minutes/week. The validity and reliability of the IPAQ was reported in a previous study involving Korean adults [18].

Height, weight, waist circumference (WC), systolic blood pressure (SBP), diastolic blood pressure (DBP), and dietary intakes of carbohydrates, fats, and proteins were assessed by trained persons. Height and weight were measured with a portable stadiometer (seca 225 stadiometer, SECA, CA, USA) and a portable scale (GL-6000-20, G-technology, Seoul, Korea), respectively, and body mass index (BMI) was calculated as weight divided by height (kg/m^2^).

Resting blood pressures were measured with a sphygmomanometer (Baumanometer® wall unit 33, Baum Co. Inc., NY, USA) with the subjects in seated position, with the arm at heart level and resting on the armrest of a chair. For resting heart rate (RHR) measurement, pulse rates at the wrist were counted in 15 s and multiplied by 4: RHR (betas/min) = the number of pulse rates in 15 s × 4. Pulse rates were counted in three separate times, and average values of the second and third counts were used. The Korean version of food frequency questionnaire with an acceptable accuracy [19] was used to assess dietary intakes of carbohydrates (g/day), fats (g/day), and proteins (g/day) in conjunction with caloric intake (kcal/day).

Appendicular skeletal muscle mass (ASM) was measured with a dual energy X-ray absorptiometry (DXA). Skeletal muscle index (SMI) was calculated by dividing the sum of ASM in the bilateral upper and lower four limbs (kg) by body weight (kg) as expressed as percentage as a modified formula from the study of Janssen et al. [20]: SMI (%) = ASM (kg) / body weight (kg) × 100. The ASM/body weight method was used previous studies involving Korean populations [21].

Fasting venous blood sampling was performed after overnight fasting to determine concentrations of glucose, total cholesterol (TC), triglycerides (TG), high-density lipoprotein cholesterol (HDL-C), insulin, hemoglobin A1c (*HbA1c*), and vitamin D. Homeostasis model assessment of insulin resistance (HOMA-IR) was calculated using a formula described by Mattew et al. [22]: fasting insulin (uU/L) x fasting glucose (nmol/L)/22.5. A detailed description of the clinical and laboratory measurements is available elsewhere [15, 16].

### Definition of metabolic syndrome

Mets was defined according to the revised National Cholesterol Education Program definition [23] with adoption of a Korean-specific WC threshold [24]. Individuals with three or more of the following criteria were defined as having Mets: 1) abdominal obesity; WC > 90 cm in men or > 85 cm in women; 2) hypertriglyceridemia; TG > 150 mg/dL or medication use; 3) low HDL-C; HDL-C < 40 mg/dL in men and < 50 mg/dL in women; 4) high resting blood pressure; SBP > 130 mmHg and/or DBP > 85 mmHg or use of antihypertensive agents; and 5) hyperglycemia; glucose > 100 mg/dL or use of anti-diabetic medication.

### Statistics

All variables were checked for normality, both visually and through the Kolmogorov-Smirnov test, and subjected to log10 transformation (i.e., TG, fasting glucose, fasting insulin, HOMA-IR, and *HbA1c*, physical activity), if necessary, prior to statistical analyses. Descriptive statistics were presented as means and standard deviations for continuous variables and as percentages for categorical variables. Analysis of variance (ANOVA) was used to test any significant differences in the measured variables between men and women or between those with and without Mets. Odd ratio (OR) and 95% confidence interval (95% CI) for Mets were calculated according to eCRF quantiles using multiple logistic regression before and after adjustments for all measured covariates. Alpha was set at 0.05. All statistical analyses were performed using the SPSS-PC statistical software (version 23.0, SPSS, Inc.).

## Results

Table 1 represents characteristics of study participants. Men had higher BMI (*p* < 0.001), waist circumference (*p* < 0.001), SMI (*p* < 0.001), physical activity (*p* < 0.001), eCRF (*p* < 0.001), resting blood pressures (*p* < 0.001), triglycerides (*p* < 0.001), fasting glucose (*p* < 0.001) and insulin (*p* < 0.001), HbA1c (*p* < 0.001), serum vitamin D (*p* < 0.001), caloric intake (*p* < 0.001), intakes of protein (*p* < 0.001), fat (*p* < 0.001), and carbohydrates (*p* < 0.001), smoking (*p* < 0.001), and heavy drinking (*p* < 0.001) but lower resting heart rate (*p* < 0.001) and HDL-C (*p* < 0.001) compared with women. No significant differences in age and HOMA-IR were observed between men and women.

**Table 1:** Descriptive statistics of study participants

Characteristic	Women (*N* = 13,400)	Men (*N* = 9885)	Total (*N* = 23,285)
Age (years)	49.1 ± 16.1	49.0 ± 15.8	49.0 ± 16.0
Body mass index (kg/m^2^)^a^	23.4 ± 3.5	24.0 ± 3.1	23.7 ± 3.3
Waist circumference (cm)^a^	78.8 ± 10.0	84.6 ± 8.9	81.2 ± 9.9
Skeletal muscle index (%)^a^	27.0 ± 2.7	34.1 ± 3.0	30.0 ± 4.5
Physical activity (METs-minutes/week)^a^	2399.8 ± 3931.1	3299.4 ± 4885.4	2781.7 ± 4384.3
Resting heart rate (beats/min)^a^	71.4 ± 9.1	70.1 ± 9.6	70.8 ± 9.3
eCRF (ml/kg/min)^a^	29.0 ± 7.8	39.6 ± 7.6	33.5 ± 9.3
Systolic BP (mmHg)^a^	116.7 ± 18.1	121.8 ± 15.9	118.8 ± 17.4
Diastolic BP (mmHg)^a^	74.3 ± 10.3	79.7 ± 10.6	76.6 ± 10.7
Total cholesterol (mg/dL)^a^	189.4 ± 36.6	187.5 ± 35.7	188.6 ± 36.3
HDL-C (mg/dL)^a^	50.8 ± 11.6	45.8 ± 10.8	48.7 ± 11.5
Triglycerides (mg/dL)^a^	115.8 ± 81.6	159.7 ± 136.7	134.5 ± 110.6
Glucose (mg/dL)^a^	96.1 ± 21.7	100.3 ± 25.3	97.9 ± 23.4
Insulin (μU/mL)^a^	10.1 ± 5.9	9.9 ± 5.6	10.0 ± 5.8
HbA1c (%)^a^	6.1 ± 1.2	6.2 ± 1.3	6.2 ± 1.3
HOMA-IR	2.5 ± 2.3	2.5 ± 1.8	2.5 ± 2.1
Vitamin D (ng/mL)^a^	17.2 ± 6.4	19.8 ± 6.9	18.3 ± 6.7
Caloric intake (kcal/day)^a^	1648 ± 641	2297 ± 922	1907 ± 829
Protein (g/day)^a^	57.9 ± 29.3	82.5 ± 43.7	67.7 ± 37.7
Fat (g/day)^a^	30.9 ± 24.1	45.3 ± 35.6	36.6 ± 30.1
Carbohydrate (g/day)^a^	285.6 ± 111.5	354.9 ± 127.1	313.2 ± 122.7
Current/past smokers, n (%)^a^	1297 (9.7)	7808 (79.0)	9105 (39.1)
Heavy drinking, n (%)^a^	619 (4.6)	2389 (24.2)	3008 (13.0)

*BP* blood pressure, *HDL-C* high-density lipoprotein cholesterol, *eCRF* non-exercise-based estimation of cardiorespiratory fitness, *HOMA* homeostatic model assessment of insulin resistance

^a^Significant difference between men and women (*p* < 0.001)

Table 2 represents the prevalence of each risk factor defining Mets. Decreased HDL-C was the most frequent risk factor, followed by hypertension, hypertriglyceridemia, hyperglycemia, and central obesity. Table 3 represents the proportion of clustering of one or more risk factors for Mets. The presence of Mets was 25.7% in the total group. Men had a higher prevalence of Mets than women (28.7% vs. 23.5%) (*p* < 0.001). Table 4 compares physical characteristics and risk factors of those with and without Mets. As expected, individuals with Mets were older, heavier, and physically less fit and had more severe profiles of risk factors compared with individuals without Mets.

**Table 2:** Prevalence of metabolic syndrome risk factors

Risk factor	Women	Men	Combined
Waist circumference^a^	26.6% (3563)	26.8% (2650)	26.7% (6213)
Fasting blood glucose^b^	23.1% (3102)	33.6% (3321)	27.6% (6423)
Blood pressure^c^	26.9% (3603)	40.3% (3988)	32.6% (7591)
HDL-C^d^	51.3% (6880)	33.3% (3292)	43.7% (10,172)
Triglycerides^e^	22.1% (2968)	38.1% (3771)	28.9% (6739)

*HDL-C* high-density lipoprotein cholesterol

^a^ > 90 for men and > 85 cm for women

^b^ > 100 mg/dL or drug treatment for impaired fasting glucose

^c^ > 130 systolic or > 85 diastolic blood pressure or drug treatment for hypertension

^d^ < 40 mg/dL for men; < 50 mg/dL for women

^e^ > 150 mg/dL or drug treatment for high serum triglycerides

**Table 3:** Clustering of metabolic syndrome risk factors

No. of risk factors meeting threshold	Women	Men	Combined
0	27.8% (3725)	22.4% (2218)	25.5% (5943)
1	29.5% (3955)	25.2% (2489)	27.7% (6444)
2	19.2% (2577)	23.7% (2344)	21.1% (4921)
3	13.8% (1845)	17.3% (1714)	15.3% (3559)
4	7.6% (1018)	9.1% (897)	8.2% (1915)
5	2.1% (280)	2.3% (223)	2.2% (503)

Individuals with three or more of the risk factors were coded for analyses as having the metabolic syndrome

**Table 4:** Individuals with metabolic syndrome (Mets) compared with apparently healthy individuals

Parameters	Individuals with Mets (*N* = 5977)	Individuals without Mets (*N* = 17,308)
Women, n (%)^a^	3143 (52.6)	10,257 (59.3)
Age (years)^a^	56.6 ± 14.0	46.4 ± 15.8
Body mass index (kg/m^2^)^a^	26.1 ± 3.2	22.8 ± 3.0
Waist circumference (cm)^a^	89.6 ± 8.1	78.4 ± 8.8
Skeletal muscle index (%)^a^	28.9 ± 4.2	30.4 ± 4.5
Physical activity (METs-minutes/week)	2716.3 ± 4354.5	2804.2 ± 4394.4
Resting heart rate (beats/min)^a^	71.6 ± 9.5	70.6 ± 9.3
eCRF (ml/kg/min)^a^	29.6 ± 9.5	34.8 ± 8.9
Systolic BP (mmHg)^a^	131.1 ± 16.6	114.6 ± 15.6
Diastolic BP (mmHg)^a^	82.6 ± 10.6	74.5 ± 10.0
Total cholesterol (mg/dL)^a^	199.1 ± 38.9	185.0 ± 34.6
HDL-C (mg/dL)^a^	41.2 ± 8.7	51.2 ± 11.3
Triglycerides (mg/dL)^a^	217.2 ± 150.0	105.9 ± 74.2
Glucose (mg/dL)^a^	112.3 ± 31.3	92.9 ± 17.3
Insulin (μU/mL)^a^	12.5 ± 8.4	9.1 ± 4.2
HbA1c (%)^a^	6.8 ± 1.4	5.8 ± 1.0
HOMA-IR^a^	3.5 ± 3.3	2.1 ± 1.2
Vitamin D (ng/mL)^a^	18.8 ± 6.7	18.1 ± 6.7
Caloric intake (kcal/day)^a^	1877.9 ± 844.4	1917.2 ± 823.2
Protein (g/day)^a^	64.7 ± 36.9	68.8 ± 37.9
Fat (g/day)^a^	32.1 ± 28.5	38.2 ± 30.4
Carbohydrate (g/day)	314.6 ± 120.9	312.8 ± 123.4
Current/past smokers, n (%)^a^	2639 (44.2)	6466 (37.4)
Heavy drinking, n (%)^a^	921 (15.5)	2087 (12.1)

Individuals with three or more of metabolic syndrome risk factors (i.e.., central obesity, hypertension, hyperglyceridemia, hypertriglyceridemia, and low HDL-C) were coded for analyses as having the metabolic syndrome (Mets)

*BP* blood pressure, *HDL-C* high-density lipoprotein cholesterol, *eCRF* non-exercise-based estimation of cardiorespiratory fitness, *HOMA* homeostatic model assessment of insulin resistance

^a^Significant difference between individuals with and without metabolic syndrome (*p* < 0.001)

Table 5 represents the results of logistic regression analyses for Mets. An inverse and graded association between eCRF and Mets was found in women (*p* < 0.001) and men (*p* < 0.001) separately. In women, the prevalence of Mets ranged from 40.0% in the lowest quantile of eCRF (quantile 1) to 14.5% in the highest quantile of eCRF (quantile 5), and the association showed a graded response, with the quantiles 2, 3, and 4 also significantly associated with a lower prevalence of the Mets compared with the quantile 1. In men, similarly, the prevalence of Mets ranged from 46.4% in the lowest quantile to 14.8% in the highest quantile, and the association showed a graded response, with the quantiles 2, 3, and 4 also significantly associated with a lower prevalence of Mets compared with the quantile 1. The lower prevalence of Mets for the highest quantile compared with the lowest quantile remained significant in both men and women even after adjustments for age (*p* < 0.001 and *p* < 0.001 in men and women, respectively) and additionally adjustments for BMI, SMI, physical activity, smoking, heavy drinking, vitamin D, caloric intakes, and intakes of macronutrients (*p* < 0.05 and *p* < 0.05 in men and women, respectively).

**Table 5:** Prevalence and logistic regression models for metabolic syndrome according to non-exercise-based estimation of cardiorespiratory fitness (eCRF) categories

Model	Quantile 1 (lowest)	Quantile 2	Quantile 3	Quantile 4	Quantile 5 (highest)
Women, No. of case	1070 (40.0)	619 (23.1)	476 (15.1)	589 (22.0)	389 (14.5)
OR	1.0	0.451^**^ (0.401–0.508)	0.325^**^ (0.286–0.368)	0.423^**^ (0.375–0.477)	0.255^**^ (0.224–0.291)
OR^a^	1.0	0.414^**^ (0.363–0.472)	0.303^**^ (0.264–0.348)	0.397^**^ (0.348–0.453)	0.231^**^ (0.200–0.267)
OR^b^	1.0	0.915 (0.761–1.100)	0.955 (0.793–1.149)	0.862 (0.714–1.041)	0.715^*^ (0.570–0.897)
OR^c^	1.0	0.952 (0.790–1.147)	0.860 (0.704–1.051)	0.862 (0.710–1.046)	0.738^*^ (0.587–0.928)
Men, No. of case	916 (46.4)	503 (25.4)	525 (26.6)	597 (30.2)	293 (14.8)
OR	1.0	0.394^**^ (0.344–0.450)	0.418^**^ (0.365–0.477)	0.499^**^ (0.438–0.569)	0.201^**^ (0.173–0.234)
OR^a^	1.0	0.397^**^ (0.346–0.454)	0.425^**^ (0.372–0.487)	0.504^**^ (0.442–0.576)	0.206^**^ (0.176–0.240)
OR^b^	1.0	0.940 (0.785–1.125)	0.863 (0.712–1.047)	0.852 (0.708–1.026)	0.730^*^ (0.585–0.911)
OR^c^	1.0	0.864 (0.707–1.056)	0.941 (0.767–1.153)	0.872 (0.710–1.072)	0.664^*^ (0.517–0.852)

*OR* odds ratio. Data are presented as OR (95% confidence interval)

Adjusted for age

Adjusted for age, body mass index, skeletal muscle index, physical activity, smoking, and heavy drinking

Adjusted for age, body mass index, skeletal muscle index, physical activity, smoking, heavy drinking, vitamin D, caloric intake, and intakes of protein, fat, and carbohydrate

^**^Significantly different compared with individuals in the lowest eCRF category (Quantile 1) at *p* < 0.001

^*^Significantly different compared with individuals in the lowest eCRF category (Quantile 1) at *p* < 0.05

## Discussion

In this population-based study, we examined the association between eCRF and Mets in Korean adults and found that eCRF was inversely associated with Mets, with men more likely to have a higher prevalence of Mets compared with women. This was the first study to show that the inverse association between eCRF and Mets remained statistically significant even after adjustments for all the covariates, implying a preventive role of CRF against the development of Mets in Korean adults.

The current findings of the study are in accordance with previous studies reporting an inverse association between CRF and Mets in Western populations. By analyzing the data obtained from two clinical trials involving 170 African-American postmenopausal women aged 40–65 years, for example, Adams-Campbel et al. [25] showed that individuals with very low CRF (< 18 mL·kg^− 1^ ·min^− 1^) had a higher prevalence of Mets, abdominal obesity, hypertriglyceridemia, and low HDL compared with individuals with moderate CRF (> 22 mL·kg^− 1^ ·min^− 1^), and the inverse association between CRF and Mets remained significant after adjustments for age, marital status, income, education, body composition, and other risk factors. By conducting a cohort of 3636 adults (1629 women) who participated in the Ball State Adult Fitness Program Longitudinal Lifestyle Study, Kelly et al. [26] also found an inverse and graded association between quartiles of CRF and Mets for both women and men, and the inverse association remained statistically significant even after adjustments for age at test date, physical activity, and cigarette smoking status. In addition, Ingle et al. [27] showed that fit British men had an approximately 50% lower prevalence of Mets compared with unfit British men, particularly in those aged 50 years or younger.

Similarly, an inverse association between CRF and Mets has been reported from previous studies involving Asian populations, such as Chinese women [28], Japanese women [29], and Chinese children [30]. In Korea, previous studies reported a significant association between physical fitness and Mets in older adults [11]. Similarly, parameters of muscular fitness and cardiopulmonary fitness were inversely and independently associated with the prevalence of Mets in older Korean adults [11]. Both high BMI and poor CRF were significantly associated with a higher prevalence of Mets in Korean adults [12], implying a synergistic effect of obesity and poor physical fitness on the etiology of Mets. In agreement with, Kim et al. [31] examined the relationship of visceral adiposity and CRF with Mets in a sample of 232 Korean overweight and obese adults, and found that high visceral adiposity and low CRF were additively associated with Mets. In that study, they also showed that high CRF alleviated the deleterious impact of visceral adiposity on Mets, implying CRF as a modifier in determining the relationship between visceral adiposity and Mets. Together, the findings of the current study agree with and extend the previous studies reporting eCRF as an independent predictor of Mets in Korean populations.

The preventive effects of higher eCRF quantiles against the prevalence of Mets observed in the present study represent levels that are attainable by most individuals. eCRF levels of approximately 29.9 mL/kg/min (or 8.5 METs) and 19.5 mL/kg/min (or 5.6 Mets) for men and women, respectively, represent the thresholds between the lowest quantile of eCRF and the second quantile of eCRF and are reasonably achievable through aerobic exercise. A lower prevalence of Mets was seen in those who were in the upper 4 quantiles in a dose-response manner, suggesting that further prevention against Mets can be achieved as one moves up the eCRF continuum with exercise training. In addition, the current findings show that logistic results for women and men were independent of all measured confounders, including age, markers of obesity and sarcopenia, physical activity, smoking, heavy drinking, vitamin D, caloric intake and intakes of macronutrients.

Several explanations can be given for the sex difference in the prevalence of Mets observed in the current study. First, we found that Korean men were more centrally obese than women. Central obesity is closely associated with whole body insulin resistance [9]. Consequently, central obesity-related insulin resistance, as shown by elevated levels of plasma insulin and HbA1c, is likely to have contributed to the sex difference. Second, an analysis by Oh et al. [32] of the 1988 KNHANES data showed that smoking was significantly associated with elevated TG and decreased HDL-C in conjunction with abdominal obesity in a dose-response manner. A higher rate of smoking might therefore have played a role in the sex difference we found. Lastly, other lifestyle risk factors such as heavy alcohol consumption, excessive caloric intakes, sarcopenic obesity, and physical inactivity may have played additional roles in determining the sex difference in the prevalence of Mets between Korean men and women [12, 21]. In particular, several mechanisms can be involved in the independent and inverse association between eCRF and Mets. First, CRF is positively associated with insulin sensitivity and/or insulin action in both overweight/obese [33] and normal subjects [34], implying its protective effect against Mets by enhancing insulin sensitivity. Second, high CRF provides a protective effect against Mets by suppressing pro-inflammation while enhancing anti-inflammation [35]. Third, high CRF alleviates the deleterious effects of central obesity and Mets [36]. Lastly, high CRF-induced promotion of mitochondrial biogenesis may lead to protection from Mets [37].

This study has several strengths and limitations. Strengths include a large sample size that is representative of Korean women and men over a wide range of age. To the best of our knowledge, this study represents the largest and Korean population-based study reporting an inverse association between eCRF and Mets after adjustment for a number of relevant confounders. In addition, the current findings support the use of eCRF as an alternative tool used to predict the risk of Mets. One study limitation is the inclusion of non-exercise-based estimates for CRF rather than objective measurements. The accuracy of eCRF used in the current study remains to be confirmed in a representative sample of Korean adults. The cross-sectional nature of the current study is another limitation in drawing conclusions about causation.

## Conclusion

The current findings confirm a protective role of eCRF against the development of Mets in Korean women and men, implying that adopting a more physically active lifestyle and promoting fitness can be clinically important in upper quantiles, especially for the lowest quantile of eCRF.

## Data Availability

Data from the Korea National Health and Nutrition Examination Surveys (KNHNES) are available from the KNHANES website (http://knhanes.cdc.go.kr). Access to data is permitted by the Korea Centers for Disease Control and Prevention (KCDC) and requests to access data may be submitted to Hyunsik Kang, PhD (hkang@skku.edu).

## Abbreviations

eCRF: non-exercise based estimation of cardiorespiratory fitness
Mets: metabolic syndrome
CVDs: Cardiovascular diseases
KNHANES: Korea National Health and Nutrition Examination Surveys
Peak VO_2_: Peak volume of oxygen consumption
HOMA-IR: Homeostasis model assessment of insulin resistance
ASM: Appendicular skeletal muscle mass
DXA: Dual energy X-ray absorptiometry
SMI: Skeletal muscle index
